# Monkeypox Virus Infection in 22-Year-Old Woman after Sexual Intercourse, New York, USA

**DOI:** 10.3201/eid2901.221662

**Published:** 2023-01

**Authors:** Nawras Zayat, Shirley Huang, Jude Wafai, Melissa Philadelphia

**Affiliations:** State University of New York Downstate Health Sciences University, Brooklyn, New York, USA (N. Zayat, S. Huang);; St. George’s University School of Medicine, West Indies, Grenada (J. Wafai);; Kings County Hospital Center, Brooklyn (M. Philadelphia)

**Keywords:** monkeypox, zoonoses, viruses, sexually transmitted infections, New York, United States

## Abstract

We report a case of a 22-year-old woman in New York, USA, who had painful vulvar and intravaginal lesions after sexual intercourse and tested positive for monkeypox virus. Literature documenting the clinical manifestations of monkeypox in female genitalia remains insufficient.

We report monkeypox virus infection in a 22-year-old woman with no remarkable medical history who sought care at the Kings County Hospital Center (Brooklyn, NY, USA) emergency department with numerous painful vulvar and intravaginal lesions. The patient reported a sexual encounter with 1 male partner 2.5 weeks before. She reported that they had vaginal sex and noted that her partner had a few dark bumps on his penis that resembled ingrown hairs. It was unknown if this partner had any sexually transmitted infections. 

Two weeks after the encounter, the patient experienced onset of myalgias, fatigue, and fever. Two days after the onset of fever, she first noticed 3 mildly painful flesh-colored bumps, which progressed the following day and became white in color and more numerous. The lesions prompted the patient to go to the emergency department at an outside hospital. At arrival, the patient was febrile to 100.7° F. She underwent screening for sexually transmitted infections, including testing for monkeypox. She was discharged home with prescriptions of lidocaine, bacitracin, and ibuprofen as needed for pain while laboratory results were pending.

On day 3 after the lesions first appeared, they became larger and more painful, to the point of causing substantial distress when the woman sat, and had spread toward her perianal region. On day 4 after the lesions’ appearance, the woman could no longer tolerate the pain and went to the emergency department at Kings County Hospital Center. At arrival, her temperature was 98.4° F, heart rate was 63 beats/min, respiratory rate was 18 breaths/min, and blood pressure was 118/72 mm Hg. Gynecologic examination revealed numerous singular, raised lesions that were umbilicated, ulcerated, vesicular, papular, and pustular, in different stages (Figure). The right labia majora was erythematous without any induration or fluctuance. We observed no lymphadenopathy on examination to the inguinal regions bilaterally. The vagina and cervix appeared normal otherwise. The uterus was small and mobile, and we noted no adnexal masses or tenderness. We observed no cutaneous lesions elsewhere in the body. Results of heart, lung, abdominal, and neurologic examinations were unremarkable.

**Figure:**
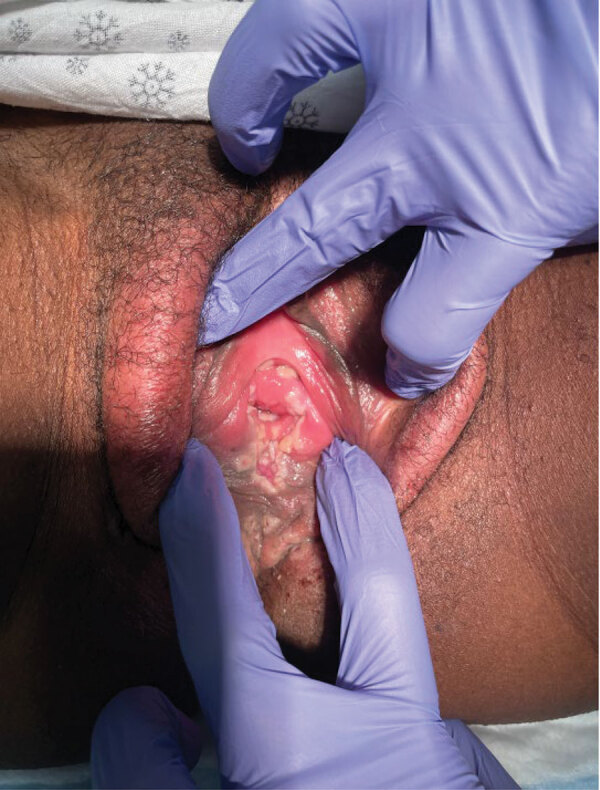
Numerous ulcerations with raised white borders extending from the labia minora into the vaginal walls in young woman with monkeypox virus infection, New York, USA

The patient was tested for gonorrhea, chlamydia, HIV, herpes simplex virus, syphilis, and monkeypox. The patient also underwent a vulvar biopsy because the gynecology team was unfamiliar with the type of genital lesions she was experiencing. While waiting for the test results, the patient was informed by her male partner that he had tested positive for monkeypox virus earlier that day. The patient was admitted to the hospital and began a 14-day course of tecovirimat (600 mg every 12 h) with accompanying isolation precautions for 21 days as per the recommendations of the physicians within Kings County Hospital’s Infectious Disease department. All results of laboratory tests were negative except for a positive PCR result for monkeypox virus. The patient continued inpatient treatment with tecovirimat and was discharged on hospital day 3 without experiencing any other complications. Approximately 10 days after the onset of lesions, the patient reported in a follow-up telehealth visit that the lesions were decreasing in size and scabbing over and that the pain had greatly subsided.

 Perineal, vaginal, and cervical lesions in women have rarely been described in the literature in the setting of a monkeypox infection. Furthermore, most cases documented from the 2022 monkeypox outbreak have primarily studied male genital lesions, with little to no mention of the clinical manifestations of monkeypox virus in female genitalia. In a literature search, we found a single case report documenting genital monkeypox lesions in a woman (*1*). In that case report, the patient experienced itchy, but painless, genital lesions extending down her labia majora that developed a week and a half after sexual intercourse with a new male partner (*1*). This patient also had a 2-day history of fever and myalgias (*1*). This patient’s lesions and symptoms were similar to those of our patient’s.

More research must be performed on the clinical manifestations of monkeypox, which would clarify the disease progression and help clinicians identify and diagnose this infection and properly treat patients. Monkeypox should be considered in the differential diagnosis of patients with genital lesions, especially in persons who are sexually active. Furthermore, there has been little study of how monkeypox virus manifests in women, especially during pregnancy, even though >400 cases have been reported in women in the United States in 2022 alone (*2*). Further consideration could elucidate the long term sequalae of monkeypox and its implications for health conditions such as pelvic inflammatory disorder, infertility, and cancer, which can occur with other sexually transmitted infections (*3*).
